# ZIF-67-Derived Co/N-Doped Carbon-Functionalized MXene for Enhanced Electrochemical Sensing of Carbendazim

**DOI:** 10.3390/molecules28217347

**Published:** 2023-10-30

**Authors:** Shuxian Chen, Jiamin Zou, Xiaowei Pan, Shaodong Zeng, Yuanjing Liu, Jianzhi Ye, Limin Lu, Shu Yang, Guoyan Zhan

**Affiliations:** 1 Laboratory of Quality and Safety Risk Assessment on Agro-Products (Zhanjiang), Ministry of Agriculture and Rural Affairs PRC, Agricultural Products Processing Research Institute, Chinese Academy of Tropical Agricultural Sciences, Zhanjiang 524001, China; 2 Key Laboratory of Crop Physiology, Ecology and Genetic Breeding, Ministry of Education, Key Laboratory of Chemical Utilization of Plant Resources of Nanchang, College of Chemistry and Materials, Jiangxi Agricultural University, Nanchang 330045, China; 3 College of Tropical Crops, Yunnan Agricultural University, Pu’er 665000, China

**Keywords:** MXene, MOFs derived carbon, carbendazim, modified electrode

## Abstract

Herein, ZIF-67-derived Co and N-doped carbon (Co/NC) particle-modified multilayer MXene (MXene@Co/NC) was developed as remarkable electrode material for carbendazim (CBZ) detection. MXene as a substrate provides an excellent conductive framework and plentiful accessibility sites. Co/NC particles embedding in MXene can not only prevent the interlayer stacking of MXene but also contribute a great deal of metal catalytic active sites and finally improve the adsorption and catalytic properties of the composite. Accordingly, the MXene@Co/NC electrode displays excellent electrocatalytic activity toward CBZ oxidation. Experimental parameters such as pH value, accumulation time, MXene@Co/NC modification volume and constituent materials’ mass ratios were optimized. Under optimal conditions, the as-prepared sensor based on MXene@Co/NC holds a broad linearity range from 0.01 μM to 45.0 μM with a low limit of detection (LOD) of 3.3 nM (S/N = 3, S means the detection signal, while N represents the noise of the instrument). Moreover, the proposed sensor displays excellent anti-interference ability, superior reproducibility, excellent stability, and successfully achieves actual applications for CBZ detection in a lettuce sample.

## 1. Introduction

Carbendazim (CBZ) is one of the systematic benzimidazole fungicides widely used in modern agricultural production, which is mainly used to prevent and control vegetable and fruit crops from being harmed by various fungi and pests [1,2]. However, CBZ accumulated in the environment and in fruits and vegetables can cause serious harm to ecosystems and the human nervous system [3]. To date, various methods have been explored for CBZ detection, such as surface-enhanced Raman spectroscopy [4,5], liquid chromatography–mass spectrometry [6], fluorimetry [7], and electrochemical methods [8,9]. Among them, electrochemical sensors stand out for their fast analysis speed, low-cost production, and ability to continuously monitor in complex systems. In particular, these properties are further enhanced when nanomaterials are incorporated into the design of electrochemical sensors. Up to now, several kinds of functional nanomaterials, such as phosphorus-doped helical carbon nanofibers [10], metal nanoparticles [11], laser-induced graphene [12] and CoCu nanoparticles modified COF/SWCNT [13], have been used as electrode modifiers for CBZ detection. Unfortunately, with the poor electrochemical activity and low residual amount of CBZ, some major issues such as low sensitivity and narrow linearity range still remain. Therefore, it is necessary to explore better sensitizing materials for electrochemical detection of CBZ.

The novel 2D transition metal carbide or nitride MXene is a promising electrode material for electrochemical sensors due to its fascinating metallic conductivity, large surface area, and satisfactory hydrophilicity [14,15]. Hitherto, MXene-based electrodes have been developed as various sensors to detect chemical and biological molecules, such as adrenaline [16], 4-chlorophenol [17], and bisphenol A [18]. In these works, the self-accumulation phenomenon for MXene nanosheets induced by hydrogen bonds and van der Waals interactions has a serious negative impact on the accessibility of electrolyte ions and electrochemical stability. To overcome this weakness, currently, a variety of materials such as metal particles [19] and carbon tubes [20] have been loaded in MXene interlamination. Meanwhile, to obtain better electrochemical sensing performance, it is necessary to explore suitable spacers that meet the anti-collapse requirements of MXene and simultaneously improve the electrocatalytic ability of the formed composites.

Metal–organic frameworks (MOFs) composed of organic ligands bridging metal ions are a recently developed intercalation material with clear porosity, large specific surface area, and certain catalytic activity [21,22,23]. Zhang et al. developed a Cr-MOF/MXene composite as a sensing substrate for xanthine detection with an acceptable linearity of 1.5–7.3 µM and 73.0–133.0 µM [24]. Li et al. prepared a ZIF-67 MOF/MXene electrode material for determination of anticancer drug flutamide with good selectivity and a wide calibration range [25]. The introduction of MOFs in MXene improves porosity and contributes to specific surface area, which is conducive to the improvement of sensing performance. Regrettably, the limited conductivity of MOFs limits the further development of nanocomposites. Metal–organic framework (MOF)-derived carbon materials have recently become the focus of electro-catalyst research on account of their rich and controllable pore structures, intrinsic heteroatom doping, and improved conductivity of precursor MOFs [26]. Typically, imidazole acid zeolite-based Co-MOF (ZIF-67)-derived carbon materials have attracted much attention. For example, Hira et al. synthesized a heteroatom-doped hierarchical porous material via ZIF-67 as a high-performance electrode for catecholamine detection, which displays a wide linearity of 5–115 µM with a low LOD of 0.43 µM [27]. Zhou et al. designed ZIF-67-derived Co nanoparticles directly supported on N-doped carbon skeletons for the sensitive determination of hydrogen peroxide [28]. Wang et al. reported MOF-derived CoPx nanoparticles embedded in hairy nitrogen-doped porous carbon (CoPx@NCNTs) polyhedrons for the electrochemical detection of p-nitrophenol with a low LOD of 0.79 nM [29]. On the one hand, the outstanding electrochemical performance of the above ZIF-67-derived carbon materials comes from the fact that ZIF-67 can maintain the original polyhedral form after carbonization and build a stable three-dimensional carbon electron conduction structure. On the other hand, the adjustable porosity and nitrogen doping enable the target analyte to be effectively fixed by physical confinement or chemisorption. The Co core could also provide a rich metal active site, which accelerates the catalytic action of the target analyte.

Herein, Co/CN derived from ZIF-67 was inserted into the interlayer of MXene as an interlayer spacer by a simple ultrasonic method, and an efficient electrochemical CBZ sensor was constructed based on the MXene@Co/NC composite. This strategy not only prevented the accumulation of MXene nanosheets, which effectively promotes electron transport, but also provided a large surface area for the distribution of Co/CN, effectively avoiding their accumulation, enabling the catalytic active site to be fully exposed and have high accessibility. Based on the synergistic effect between MXene and Co/NC, the electrochemical CBZ sensor displayed excellent analytical performance with high sensitivity, splendid stability, and good reproducibility for CBZ detection.

## 2. Results and Discussion

### 2.1. Characterization

**Figure 1:**
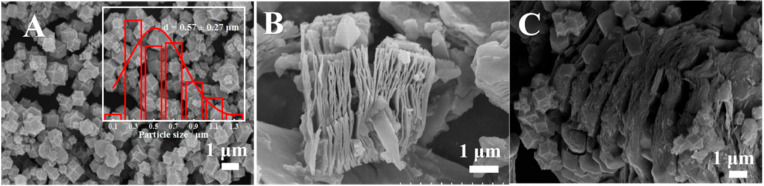
SEM images of Co/NC, insert is the particle size statistical analysis of Co/NC (**A**), MXene (**B**), MXene@Co/NC (**C**).

SEM was applied to verify the micromorphologies of Co/NC, MXene, and MXene@Co/NC. As shown, Co/NC (Figure 1A) displays a polyhedral morphology with a wrinkled surface. The particle size of Co/NC was statistically analyzed via Nano Measurer, in which the minimum particle size is 0.14 μm and the average particle size is 0.57 μm (inset of Figure 1A). MXene presents the representative delaminated structure (Figure 1B). According to the SEM of the MXene@Co/NC composite (Figure 1C), it is clearly seen that a part of the small-size Co/NC particles are well embedded between the fluffy MXene interlayer and some large-size particles are attached to the edge of the MXene. The unique structure could provide a large utilization space, expose more active sites, and ultimately increase catalytic activity. Moreover, the energy-dispersive spectroscopy (EDS) mapping images show that the Co (Figure S1A), N (Figure S1B), and Ti (Figure S1C) elements are uniformly distributed on the MXene@Co/NC surface, indicating the successful combination of Co/NC with Mxene.

**Figure 2:**
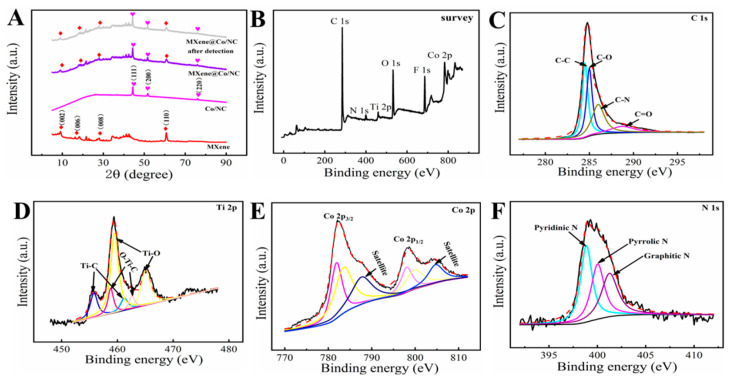
(**A**) XRD spectra for Co/NC, MXene, MXene@Co/NC, and MXene@Co/NC after detection; (**B**) XPS survey spectra of MXene@Co/NC; the C 1s (**C**), Ti 2p (**D**), Co 2p (**E**), and N 1s (**F**) core-level spectra of MXene@Co/NC.

The crystallographic structure of Co/NC, MXene, and MXene@Co/NC were proved by XRD analysis (Figure 2A). In the diffract gram of MXene, the diffraction peaks positioned at 9.1°, 18.4°, 28.0°, and 60.8° match the (002), (006), (008), and (110) planes of MXene. For the Co/NC, the diffraction peaks at 44.3°, 51.6°, and 76.0° match the (111), (200), and (220) crystal planes of the Co particle, which are consistent with previous reports [30]. The XRD pattern of MXene@Co/NC simultaneously presents the typical peaks of Co/NC and primary characteristic peaks of MXene, confirming that the MXene@Co/NC nanohybrid was successfully constructed. Furthermore, compared with the MXene@Co/NC, the diffraction peak of the MXene@Co/NC after detection has no obvious change, which means that the crystal structure of the MXene@Co/NC after the sensing process remains stable.

Figure S2A reveals the N_2_ adsorption/desorption isotherms of the MXene@Co/NC at 77 k. It displays that the specific surface area of MXene@Co/NC is 239.5 m^2^ g^−1^ and there is a typical type I curve, indicating the presence of numerous micropores [31].

XPS was employed to investigate the elemental composition and chemical state analysis of the MXene@Co/NC composite. As shown in Figure 2B, the XPS survey spectra of MXene@Co/NC manifest that the composite contains C, N, Ti, O, F, and Co elements. According to the C 1s high-resolution spectrum (Figure 2C), four peaks are positioned at 284.51 eV, 285.00 eV, 286.00 eV, and 288.71 eV, which are indexed in C-C, C-O, C-N, and O=C, respectively. The XPS spectra of the Ti 2p peak (Figure 2D) consist of three pairs of spin orbital peaks, including Ti-C 2p_3/2_/Ti-C 2p_1/2_ (455.79 eV/461.22 eV), O-Ti-C 2p_3/2_/O-Ti-C 2p_1/2_ (458.80 eV/462.64 eV), and Ti-O 2p_3/2_/Ti-O 2p_1/2_ (459.50 eV/465.09 eV). In the Co 2p high-resolution section (Figure 2E), two pairs of strong peaks located at 781.88 eV/798.03 eV and 783.50 eV /799.84 eV can well match Co^3+^ 2p^3/2^/Co^3+^ 2p^1/2^ and Co^2+^ 2p_3/2_/Co^2+^ 2p_1/2_ [32]. Meanwhile, there are two satellite peaks located at 787.65 and 804.73 eV. The high-resolution spectra of N 1s (Figure 2F) is a deconvolution of three parts, including pyridine N (398.87 eV), pyridine N (400.03 eV), and graphite N (401.26 eV) [33].

### 2.2. EIS Characterization

**Figure 3:**
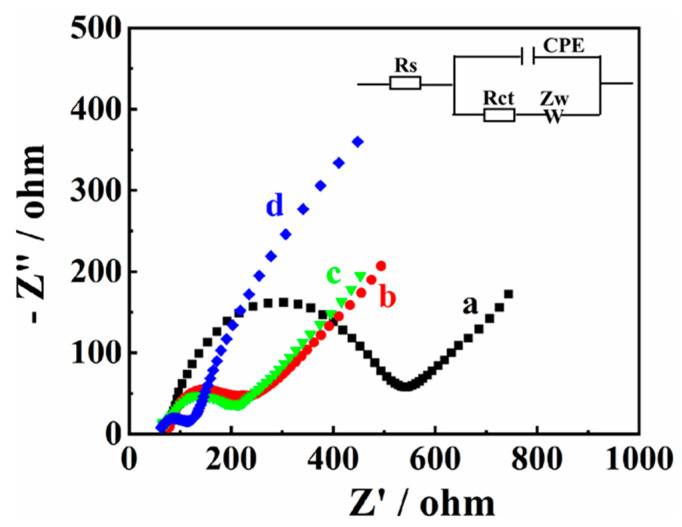
Nyquist plots of bare GCE (a), MXene/GCE (b), Co/NC/GCE (c), and MXene@CO/NC/GCE (d) in 5 mM [Fe(CN)_6_]^3−/4−^ solution containing 0.1 M KCl.

Electrochemical impedance spectroscopy (EIS) was employed to research the surface impedance of the modified electrode. The Nyquist curves of bare GCE (a), MXene/GCE (b), Co/NC/GCE (c), and MXene@Co/NC/GCE (d) are exhibited in Figure 3. Impedance data are simulated using associated equivalent circuits, which involves the constant phase element (CPE), active electrolyte resistance (Rs), Warburg impedance (Zw), and charge transfer resistance (Rct).

According to the fitted data, the Rct values of bare GCE (a), MXene/GCE (b), and Co/NC/GCE (c) are calculated as 483.3 Ω, 181.4 Ω, and 169.9 Ω, respectively. The Rct values of MXene/GCE and Co/NC/GCE are lower than bare GCE, signifying the admirable electrical conductivity of MXene and Co/NC/GCE. In addition, the lowest Rct value (80.7 Ω) is obtained at MXene@Co/NC/GCE, testifying that the combination of MXene and Co/NC can reduce the electron transfer resistance.

### 2.3. Electrochemical Behavior of CBZ at the Modified Electrodes

**Figure 4:**
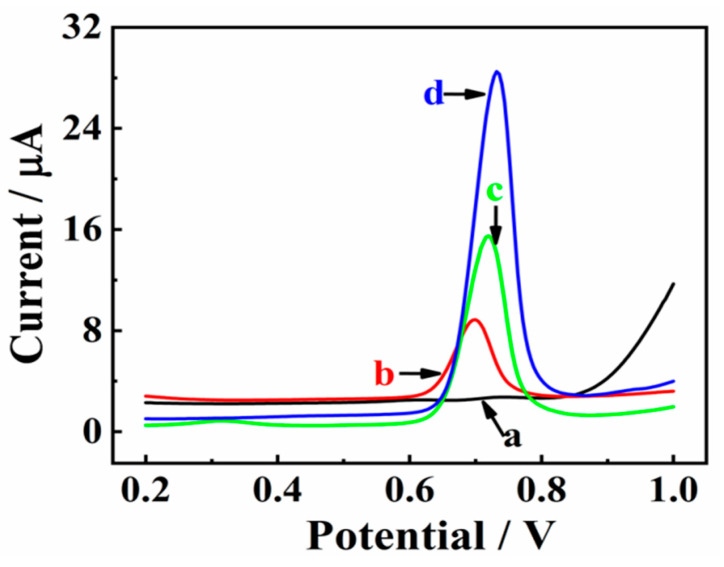
DPV behaviors of CBZ (25.0 μM) in 0.1 M PBS (pH 7.0) on bare GCE (a), MXene/GCE (b), Co/NC/GCE (c), and MXene@Co/NC/GCE (d).

The electrochemical behaviors of CBZ (25.0 μM) at bare GCE (a), Mxene/GCE (b), Co/NC/GCE (c), and MXene@Co/NC/GCE (d) were recorded by DPV in 0.1 M PBS (pH 7.0). As displayed in Figure 4, the faintest oxidation peak appears at bare GCE (curve a). However, MXene/GCE (b) shows significant enhancement in the oxidation peak current, which could be due to the satisfactory conductivity of MXene/GCE. Furthermore, the oxidation peak at Co/NC/GCE (c) becomes much stronger, which is ascribed to abundant Co active sites and the three-dimensional carbon electron conduction structure of Co/NC/GCE. The highest oxidation peak is found at MXene@Co/NC/GCE (d). This splendid performance is mainly attributed to the synergistic effect of Co/NC and MXene. Co/NC particles embedded in MXene nanosheets prevent MXene layer accumulation and reasonably increase the available active space. MXene@Co/NC with a hierarchical architecture and good electrical conductivity provides an open mass transfer channel and facilitates electron transfer to improve electrocatalytic performance.

### 2.4. Optimization of Analytical Conditions

#### 2.4.1. Effect of the Ratio of MXene and Co/NC

**Figure 5:**
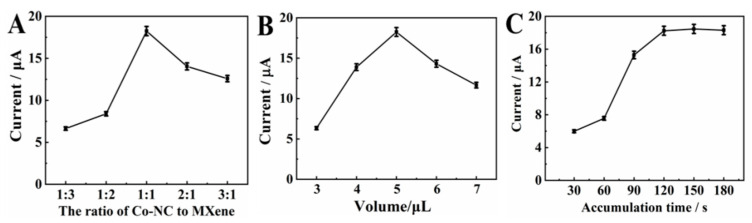
Influence of (**A**) the ratio of Co-NC to MXene/GCE; (**B**) influence of the suspension volume of Co-NC/MXene/GCE; (**C**) effect of accumulation time with a deposition potential in 0.1 M PBS (pH 7.0) with addition of 20.0 μM CBZ.

The effect of the ratio of MXene and Co/NC on the current response of CBZ was studied. The error bar and measurement data are provided in Table S1. Figure 5A shows the relationship between the current response of CBZ and the ratio of MXene and Co/NC. When the ratio of Co/NC:MXene increases from 1:3 to 1:1, the current response is significantly increased, which confirms that the increase in the proportion of Co/NC particles helps to promote the electrocatalytic ability of the composite. However, the peak current decreases with the ratio of Co/NC:MXene from 1:1 to 3:1, meaning that excessive Co/NC particles dispersed among MXene can affect the mass transfer channel. Therefore, the optimal ratio of Co/NC:MXene is 1:1.

#### 2.4.2. Effect of the Volume of MXene@Co/NC on GCE

The effect of MXene@Co/NC modification volume on the current response of CBZ was studied. The error bar and measurement data are provided in Table S1. Figure 5B shows the relationship between the oxidation peak current of CBZ and the MXene@Co/NC modification volume on GCE. It can be clearly seen that from 3.0 μL to 5.0 μL, the oxidation peak current rises in turn. This is attributed to the fact that more active sites are generated along with an increased amount of MXene@Co/NC on the GCE. Nevertheless, the peak current drops sharply after 5.0 μL. This phenomenon is on account of the excess MXene@Co/NC on the electrode surface leading to the coverage of partial active sites. Therefore, the optimal modified volume of MXene@Co/NC is 5.0 μL.

#### 2.4.3. Effect of Accumulation Time

The adherence amount of CBZ on MXene@Co/NC/GCE can be increased by prolonging accumulation time. The error bar and measurement data are provided in Table S1. In general, the large adhesion amount of the analyte can acquire a great current response. Figure 5C describes the correlation as regards the accumulation time and the oxidation peak current. As the accumulation time changes from 30 s to 120 s, the current response enhances gradually, whereas the current nearly stops changing with the extension of the accumulation time, signifying that the absorption of CBZ on the MXene@Co/NC/GCE surface is close to the saturation state at 120 s. In order to take into account the efficiency and sensitivity of detection, 120 s is selected as the most suitable accumulation time.

#### 2.4.4. Effect of pH

**Figure 6:**
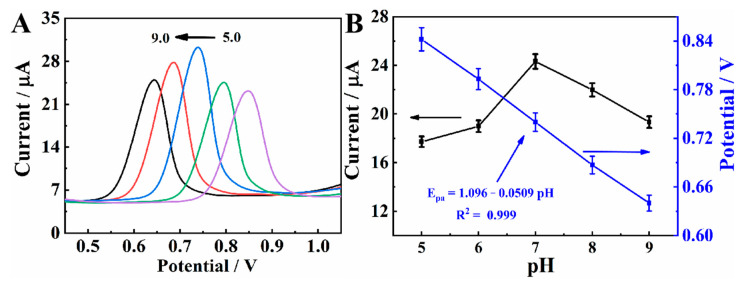
(**A**) DPVs of MXene@Co/NC/GCE in PBS with different pH containing 25.0 μM CBZ. (**B**) Effect of pH value on the E_pa_ and I_pa_.

The effect of buffer pH on the electrochemical behavior of MXene@Co/NC/GCE at 25.0 μM CBZ was studied by DPV. The error bar and measurement data are provided in Table S2. As shown in Figure 6A, the oxidation peak potential shifts toward a negative direction as pH increases, verifying that protons are related to the electrode reaction. As can be seen from Figure 6B, oxidation peak potential has a good linearity with pH. The slope of −0.0509 V is approximately the theoretical value of −0.059 V, signifying that the number of protons involved in the electrode reaction is the same as the number of electrons transferred [9]. Obviously, the peak current achieves its maximum at pH 7.0. In the follow-up experiment, PBS with pH of 7.0 is used as the buffer solution.

### 2.5. Kinetics Studies

**Figure 7:**
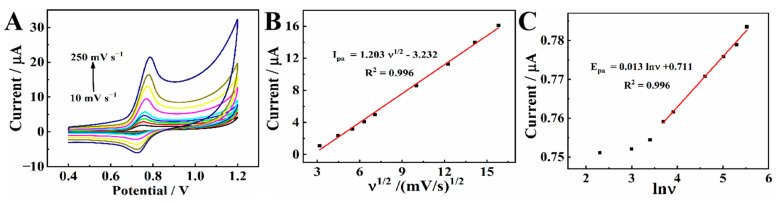
(**A**) CVs of 20.0 μM CBZ on MXene@Co/NC/GCE in 0.1 M PBS (pH 7.0) at different scan rates: 10, 20, 30, 40, 50, 100, 150, 200, and 250 mV s^−1^; (**B**) linear relationship between the peak current (I_pa_) and the ν^1/2^; (**C**) the relationship between potential (E_pa_) and ln ν.

To figure out the electrochemical reaction kinetics of CBZ, CVs of CBZ were investigated with a range of scanning rates (v) on MXene@Co/NC/GCE. As shown in Figure 7A, E_pa_ shifts toward a positive direction as the scanning rate increases. From 10 to 250 mV s^−1^ (Figure 7B), the current gradually increases. There is a good linear correlation between the peak current and ν^1/2^. The linear equation is I_pa_ (μA) = 1.203 ν^1/2^ − 3.232 (R^2^ = 0.996), indicating that the electrochemical reaction of CBZ on MXene@Co/NC/GCE is a diffusion-controlled process [34].

As shown in Figure 7C, with high scanning rates, the peak potential is linearly related to the natural logarithm of the scanning rate. The correlation formula is E_pa_ (V) = 0.013 lnν + 0.711 (R^2^ = 0.996). This is on the basis of Butler–Volmer equation [35]:

(1) $$E_{\mathrm{pa}}=E^{0\prime }+\frac{{\mathrm{RTk}}_{s}}{2\alpha \mathrm{nF}}\;\ln\frac{{\mathrm{RTk}}_{s}}{\alpha \mathrm{nF}}+\frac{\mathrm{RT}}{2\alpha \mathrm{nF}}\;\mathrm{lnv}$$

**Scheme 1:**
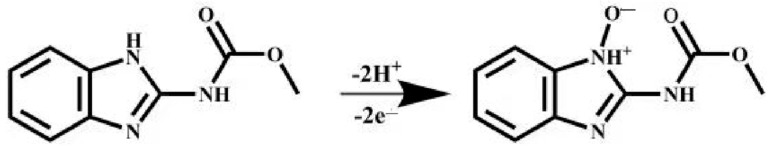
The mechanism for electrochemical process of CBZ.

Here, R, T, and K are counted as the gas constant, absolute temperature, and standard rate coefficient, respectively. F means Faraday constant. α means the electron transfer coefficient, which is usually counted as 0.5. Hence, n is estimated to be 2. From the fact that the number of electrons is consistent with the number of transferred protons, it can be considered that two electrons and two protons participate in the oxidation reaction of CBZ at MXene@Co/NC/GCE (Scheme 1).

### 2.6. Detection of CBZ at MXene@Co/NC/GCE

**Figure 8:**
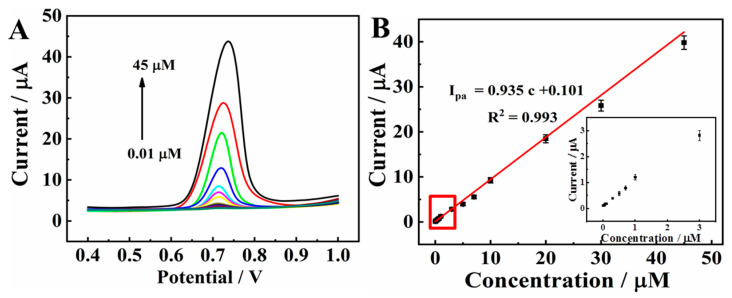
(**A**) DPVs of 0.01, 0.03, 0.05, 0.07, 0.1, 0.3, 0.5, 0.7, 1.0, 3.0, 5.0, 7.0, 10.0, 20.0, 30.0, and 45.0 μM CBZ on MXene@Co/NC/GCE in 0.1 M PBS (pH 7.0); (**B**) linear curve of oxidation peak current vs. concentration, insert is the enlarged view of lower CBZ concentration.

Figure 8A displays the DPV signal curves of CBZ with a series of concentrations in 0.1 M PBS (pH 7.0) on MXene@Co/NC/GCE. The current proportionally increases as the CBZ concentration changes from 0.01 μM to 45.0 μM. As displayed in Figure 8B, the peak current and c present a good linearity with the regression formula of I_pa_ = 0.935 c + 0.101 (R^2^ = 0.993). The limit of detection (LOD) is counted as 3.3 nM (S/N = 3), which is calculated using the following equation [36,37]:

LOD = 3 × SD/S(2)

where SD means the standard deviation of blank signal and S denotes the sensitivity of the method (equal to the slope of the calibration curve). The electrochemical sensing performance of MXene@Co/NC/GCE was further compared with previously reported electrochemical CBZ sensors (shown in Table 1). The results showed that the MXene@Co/NC/GCE possessed a wider linear range and lower LOD than the other sensors, including graphene-based sensing platforms [12,38], carbon nanotube-based sensing platforms [39,40], AuNPs/BC/GCE [41], and In_2_S_3_/GCE [42], which might be attributed to the synergetic effect between the large surface area, excellent catalytic performance, and rapid electronic transfer of MXene@Co/NC.

### 2.7. Interferences, Reproducibility, Repeatability, and Long-Term Stability of MXene@Co/NC/GCE

**Figure 9:**
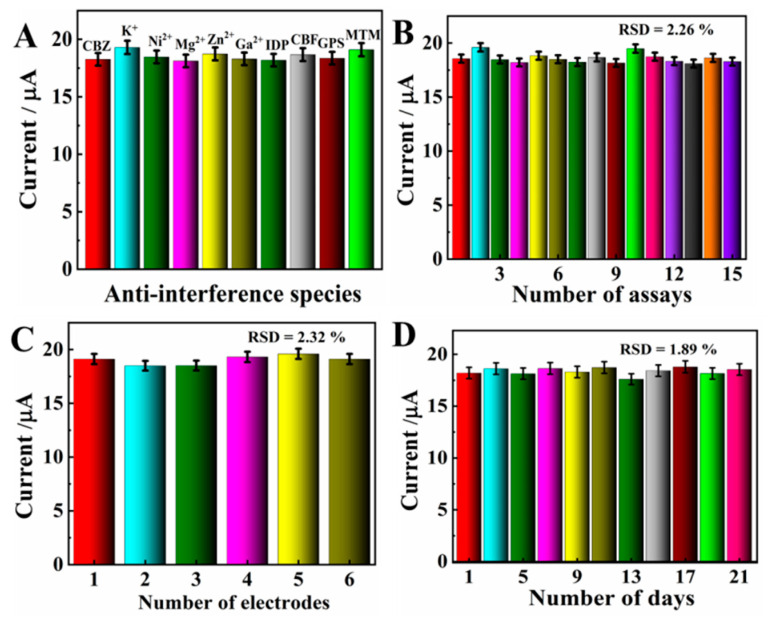
(**A**) Anti-interference studies of MXene@Co/NC/GCE. (**B**) DPV was repeated 15 times for MXene@Co/NC/GCE at 100 mV s^−1^ in PBS solution containing 20.0 μM CBZ. (**C**) Repetitive DPV responses of MXene@Co/NC/GCE with the same electrode. (**D**) DPV responses of MXene@Co/NC/GCE toward 20.0 μM CBZ in 21 days.

The anti-interference of MXene/NG/GCE was evaluated by measuring the DPV response of 20.0 μM CBZ with a variety of interferents. The experimental results show that all signal changes were less than 5%, which verifies 100-fold concomitant ions (K^+^, Ni^2+^, Mg^2+^, Zn^2+^, and Ca^2+^) and 10-fold interferents including imidacloprid (IDP), glyphosate (GPS), carbofuran (CBF), and methomyl (MTM), showing no obvious interferences with CBZ (Figure 9A). Accordingly, the MXene@Co/NC/GCE possesses gratifying selectivity.

To affirm the repeatability of the proposed electrode, MXene@Co/NC/GCE was employed to perform 15 consecutive tests for 20.0 μM CBZ. The result shows that the relative standard deviation (RSD) is 2.26% (Figure 9B). Meanwhile, six parallel electrodes were used to measure the response current of 20.0 μM CBZ under consistent experiment conditions (Figure 9C). The RSD of the response current is 2.32%. These values verify that MXene@Co/NC/GCE possesses pleasing repeatability and reproducibility.

Furthermore, to prove the long-term stability of MXene@Co/NC/GCE, the DPV response for 20.0 μM CBZ on MXene@Co/NC/GCE was monitored every 2 days (Figure 9D). After 21 days, the changes in peak current were below 5.0%, certifying the preeminent long-term stability of MXene@Co/NC/GCE.

### 2.8. Real Sample Analysis

To evaluate the practicability of the as-prepared sensing platform, MXene@Co/NC/GCE was used for the detection of CBZ in lettuce samples with the standard addition method (Table 2). The hydroponic lettuce purchased at Zhanjiang farmers market was crushed into pulp and filtered with 0.45μm nylon filter membrane. The filtered solution was diluted 100 times with 0.1 M PBS (pH 7.0). Subsequently, a specific concentration of CBZ (0 µM, 1.0 µM, 5.0 µM, 20.0 µM) was appended to the diluted sample. These samples were detected by DPV on MXene@Co/NC/GCE. The detailed DPV diagram is shown in Figure S3. As displayed in Table 2, the recoveries are 97.95~101.60% and the range of the RSD obtained by the calculation is 2.17~2.22%, certifying that MXene@Co/NC/GCE can be applied to the practical sample detection of CBZ.

## 3. Materials and Methods

### 3.1. Reagents

CBZ were acquired from Zhanjiang Yuetai Reagent Instrument Co., Ltd. MXene was supplied from Nanjing Mingshan new material Technology Co., Ltd. (Nanjing, China) Co(NO_3_)·6H_2_O and 2-Methylimidazole were obtained from Guangzhou Chemical reagent Factory. NaH_2_PO_3_ (99%) and Na_2_HPO_3_ (99%) were purchased from Shanghai Macklin Biochemical Technology Co., Ltd. (Shanghai, China). In this work, an analytical reagent was used to perform the experiment. Deionized water was used to support the experiment.

### 3.2. Apparatuses

Scanning electron microscopy (SEM, Hitachi Company, Tokyo, Japan) was used to observe the morphology of the materials. X-ray diffraction (XRD, smartlab9, Rigaku Corporation, Tokyo, Japan) and X-ray photoelectron spectroscopy (XPS, Escalab 250Xi, Thermo Fisher Scientific, Waltham, MA, USA) were employed to observe the component structure of the compound. Electrochemical data were measured at an electrochemical workstation (CHI660E, Shanghai, China) using a typical three-electrode system, which is made up of the opposite electrode (Pt electrode), the reference electrode (saturated calomel electrode), and the working electrode (bare or modified GCE).

### 3.3. Preparation of the Co/NC

Co/NC materials were prepared according to the following steps. Firstly, 1.0 mmol Co(NO_3_)_2_·6H_2_O was dissolved in 25 mL methanol, followed by the addition of 4.0 mmol 2-dimethylimidazole. Subsequently, the aqueous dispersion mixture was vigorously stirred for 0.5 h and left to stand for 24 h to acquire ZIF-67. ZIF-67 was centrifuged, washed, and dried at 60 °C for 12 h under the vacuum condition. Finally, the ZIF-67 was annealed at 800 °C for 2 h under a N_2_ atmosphere to obtain Co/NC.

### 3.4. Preparation of the MXene@Co/NC Composite

The MXene@Co/NC composite was prepared with an ultrasound-assisted self-assembly method. Typically, MXene (10.0 mg) was uniformly dispersed in DMF (20 mL) and sonicated for 10 min. Co/NC (10.0 mg) was then added to the above suspension under ultrasound stirring for 30 min. Finally, the black suspensions were centrifuged and the pellets were washed three times with deionized water and absolute alcohol. The as-prepared product was named MXene@Co/NC after being dried in a vacuum at 60 °C.

### 3.5. Fabrication of Modified Electrodes

Firstly, the glassy carbon electrode (GCE) was perpendicularly polished on the chamois leather with a 0.5 μm alumina slurry. Subsequently, the polished GCE was ultrasonically cleaned in deionized water and ethanol successively.

**Scheme 2:**
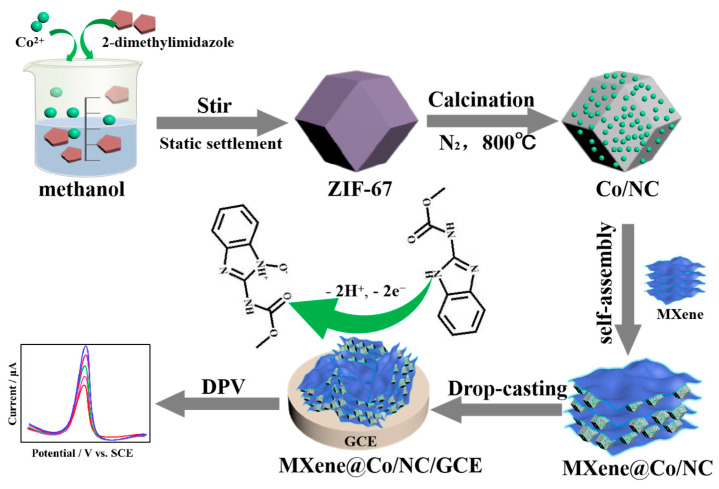
The synthesis process of MXene@Co/NC/GCE and its application for CBZ detection.

To construct MXene@Co/NC/GCE, 5.0 μL of the MXene@Co/NC suspension (1 mg mL^−1^) was coated onto the GCE and then dried for later use. Co/NC/GCE and MXene/GCE were prepared under the analogical procedure. The manufacture process of MXene@Co/NC/GCE and its application for CBZ detection are shown in Scheme 2.

### 3.6. Electrochemical Method

Electrochemical measurements were performed in 5.0 mL 0.1 M phosphate buffer solution (PBS) including quantificational CBZ. Differential pulse voltammetry (DPV) was measured by scanning the potential range from 0.4 to 1.0 V with a 0.004 V Incr E, 0.5 sec pulse period, and 0.05 V amplitude. Electrochemical impedance spectroscopy (EIS) was performed in 5.0 mM [Fe(CN)_6_]^3−/4−^ (1:1) including 0.1 M KCl; the frequency range was 1 Hz to 100 kHz and the amplitude was 0.005 V. Cyclic voltammetry (CV) was conducted with a potential scan at 0.4 V–1.2 V and a scan rate of 100 mV s^−1^.

## 4. Conclusions

In summary, a high-efficiency electrochemical sensing platform for CBZ was proposed based on MXene@Co/NC. Specifically, Co/NC was obtained with ZIF-67 as precursor and inherited its unique merits such as the three-dimensional structure, high porosity, and large specific surface area. Then, the Co/NC was inserted into the interlayer of MXene as an interlayer spacer, which not only effectively avoided the stacking of MXene nanosheets but also improved the dispersion of Co/NC, endowing the composite with rapid electron transport and an excellent catalytic property. Under the optimal conditions, MXene@Co/NC/GCE displayed a wide linear range (0.01–45.0 μM) and low LOD of 3.3 nM for CBZ. Moreover, the developed electrochemical sensor exhibited outstanding stability, repeatability and satisfactory recovery in lettuce samples, signifying that MXene@Co/NC is a prospective material for the construction of pesticide electrochemical sensors.

## Figures and Tables

**Table 1:** Comparison of different modified electrodes used for the detection of CBZ.

Modified Electrodes	Liner Range (μM)	LOD (nM)	Methods	Reference
LIG/Pt	1–40	670	DPV	[12]
G-PtNi/3D-NPC	0.5–30	0.04	DPV	[38]
MWCNTs-COOH/BP	0.009–1	4.0	DPV	[39]
Ce-MOFs@MWCNTs@ ZnO/GCE	0.05–10 and 10–50	13.2	DPV	[40]
AuNPs/BC/GCE	0.01–900	4.4	DPV	[41]
In_2_S_3_/GCE	5–80	670	DPV	[42]
MXene@Co/NC/GCE	0.01–45	3.3	DPV	This work

LIG: laser-induced graphene/PtNPs; MWCNTs-COOH: carboxylated multiwalled carbon nanotubes; BP: black phosphorus; AuNPs: gold nanoparticles; BC: bioderived carbon; G: graphene; 3-D NPC: three-dimensional N-doped porous carbon.

**Table 2:** Determination of CBZ in lettuce samples.

Samples	Added (μM)	Found (μM)	Recovery (%)	RSD (%)
1	0.000	-	-	-
2	1.000	0.980 ± 0.026	97.95	2.17
3	5.000	5.083 ± 0.138	101.60	2.22
4	20.000	19.840 ± 0.539	99.20	2.21

## Data Availability

The data presented in this study are available in the article.

## Supplementary Materials

The following supporting information can be downloaded at: https://www.mdpi.com/article/10.3390/molecules28217347/s1.
