# Supplementary material for: ZIF-67-Derived Co/N-Doped Carbon-Functionalized MXene for Enhanced Electrochemical Sensing of Carbendazim
Source: Molecules. 2023 Oct 30;28(21):7347. doi: 10.3390/molecules28217347 (PMC10650760; doi:10.3390/molecules28217347)
Supplement: Supplementary file 1 [file molecules-28-07347-s001.zip › molecules-2625695-supplementary.pdf]

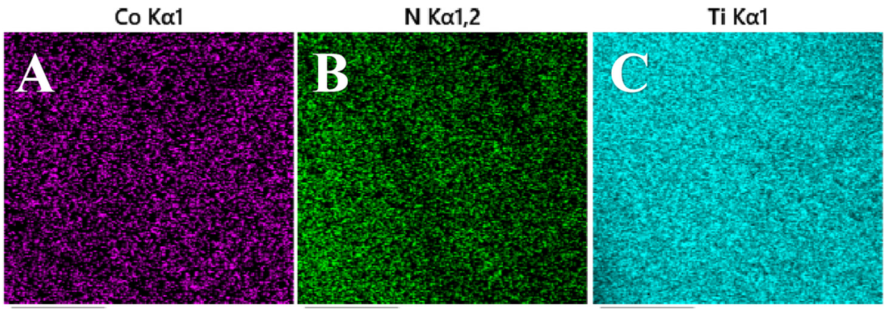

Figure S1. EDS mapping images of Co (**A**), N (**B**) and Ti (**C**) elements in MXene@Co/NC.

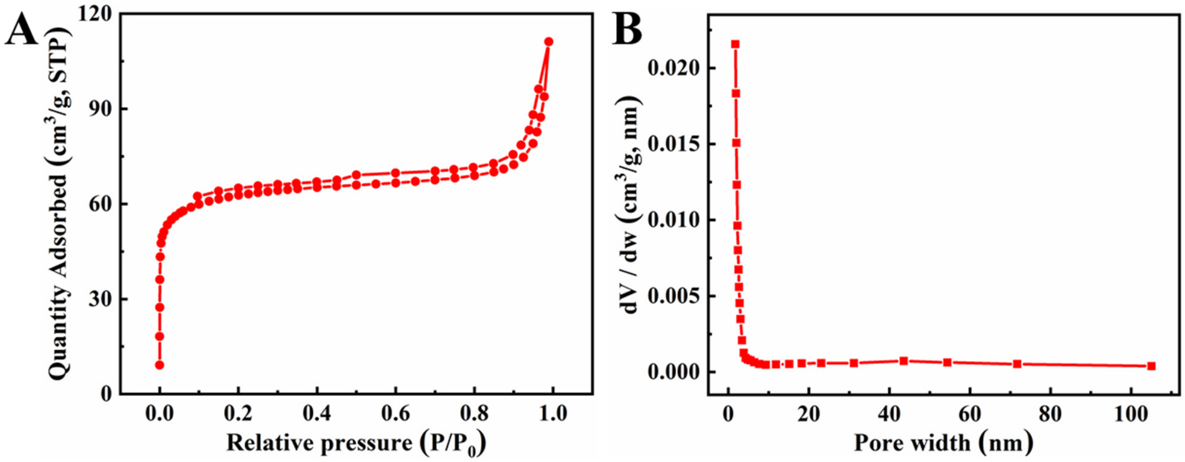

Figure S2. (**A**) Nitrogen adsorption/desorption isotherms and (**B**) pore size distributions of Mxene@Co/NC.

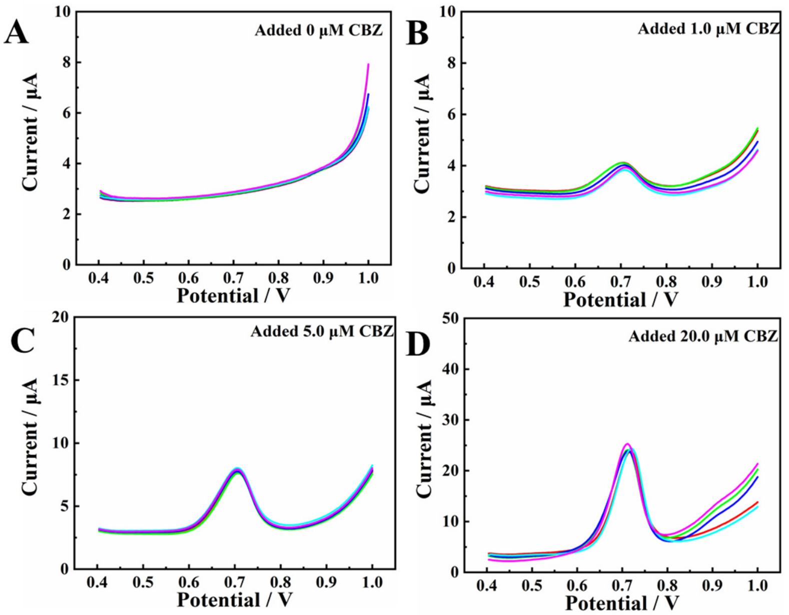

Figure S3. The DPV data of real sample analysis with the addition of 0 μM CBZ (**A**), 1.0 μM CBZ (**B**), 5.0 μM CBZ (**C**) and 20.0 μM CBZ (**D**).

Table S1. The error bar and measurement data of influence of the ration of Co-NC to MXene/GCE, the suspension volume of Co-NC/MXene/GCE and accumulation time.

Ration	y axis Current (μA)	Volume (μL)	y axis Current (μA)	accumulation time (s)	Current (μA)
1:3	6.636±0.199	3	6.331±0.189	30	5.992±0.179
1:2	8.396±0.252	4	13.891±0.417	60	7.560±0.227
1:1	18.240±0.547	5	18.242±0.547	90	15.291±0.458
2:1	14.040±0.421	6	14.310±0.429	120	18.240±0.547
3:1	12.580±0.377	7	11.661±0.350	150	18.461±0.553
-	-	-	-	180	18.310±0.549

Table S2. The error bar and measurement data of the effect of pH value.

pH	y axis Current (μA)	y axis Potential (V)
5	17.710±0.442	0.842±0.013
6	18.981±0.474	0.793±0.012
7	24.321±0.608	0.740±0.011
8	21.981±0.549	0.687±0.011
9	19.330±0.483	0.640±0.010
